# Electronic Source Data Transcription for Electronic Case Report Forms in China: Validation of the Electronic Source Record Tool in a Real-world Ophthalmology Study

**DOI:** 10.2196/43229

**Published:** 2022-12-16

**Authors:** Bin Wang, Junkai Lai, Mimi Liu, Feifei Jin, Yifei Peng, Chen Yao

**Affiliations:** 1 Peking University Clinical Research Institute Peking University First Hospital Beijing China; 2 Institute of Automation Chinese Academy of Sciences Beijing China; 3 School of Ophthalmology and Optometry Eye Hospital of Wenzhou Medical University Wenzhou China; 4 Trauma Medicine Center Peking University People's Hospital Beijing China; 5 Key Laboratory of Trauma treatment and Neural Regeneration Peking University Ministry of Education Beijing China; 6 National Center for Trauma Medicine of China Beijing China; 7 Hangzhou Tigermed Consulting Co, Ltd Hangzhou China; 8 Hainan Institute of Real World Data Qionghai China

**Keywords:** electronic medical record, electronic health record, electronic source, eSource, eSource record tool, real-world data, data transcription, data quality, System Usability Scale, ophthalmology

## Abstract

**Background:**

As researchers are increasingly interested in real-world studies (RWSs), improving data collection efficiency and data quality has become an important challenge. An electronic source (eSource) generally includes direct capture, collection, and storage of electronic data to simplify clinical research. It can improve data quality and patient safety and reduce clinical trial costs. Although there are already large projects on eSource technology, there is a lack of experience in using eSource technology to implement RWSs. Our team designed and developed an eSource record (ESR) system in China. In a preliminary prospective study, we selected a cosmetic medical device project to evaluate ESR software’s effect on data collection and transcription. As the previous case verification was simple, we plan to choose more complicated ophthalmology projects to further evaluate the ESR.

**Objective:**

We aimed to evaluate the data transcription efficiency and quality of ESR software in retrospective studies to verify the feasibility of using eSource as an alternative to traditional manual transcription of data in RWS projects.

**Methods:**

The approved ophthalmic femtosecond laser project was used for ESR case validation. This study compared the efficiency and quality of data transcription between the eSource method using ESR software and the traditional clinical research model of manually transcribing the data. Usability refers to the quality of a user’s experience when interacting with products or systems including websites, software, devices, or applications. To evaluate the system availability of ESR, we used the System Usability Scale (SUS). The questionnaire consisted of the following 2 parts: participant information and SUS evaluation of the electronic medical record (EMR), electronic data capture (EDC), and ESR systems. By accessing log data from the EDC system previously used by the research project, all the time spent from the beginning to the end of the study could be counted.

**Results:**

In terms of transcription time cost per field, the eSource method can reduce the time cost by 81.8% (11.2/13.7). Compared with traditional manual data transcription, the eSource method has higher data transcription quality (correct entry rate of 2356/2400, 98.17% vs 47,991/51,424, 93.32%). A total of 15 questionnaires were received with a response rate of 100%. In terms of usability, the average overall SUS scores of the EMR, EDC, and ESR systems were 50.3 (SD 21.9), 51.5 (SD 14.2), and 63.0 (SD 11.3; contract research organization experts: 69.5, SD 11.5; clinicians: 59.8, SD 10.2), respectively. The Cronbach α for the SUS items of the EMR, EDC, and ESR systems were 0.591 (95% CI −0.012 to 0.903), 0.588 (95% CI −0.288 to 0.951), and 0.785 (95% CI 0.576-0.916), respectively.

**Conclusions:**

In real-world ophthalmology studies, the eSource approach based on the ESR system can replace the traditional clinical research model that relies on the manual transcription of data.

## Introduction

### Background

An electronic source (eSource) generally includes the direct capture, collection, and storage of electronic data (eg, electronic medical records [EMRs], electronic health records [EHRs], or wearable devices) to simplify clinical research [1]. It can improve data quality and patient safety and reduce clinical trial costs. Despite the existence of several United States Food and Drug Administration guidelines [1,2] and European Medicines Agency guidelines [3], the development, implementation, and evaluation of EMR-specific electronic resource solutions are limited. Owing to known challenges such as the limited interoperability of EMRs and electronic data capture (EDC) systems, unstructured data (eg, researcher notes or comments), and the need for some data (eg, research-specific data not included in the EMR) to be manually transcribed and treated, accessing and correcting the source data in real time during data collection can be slow [4]. The direct use of EHR data in clinical research helps to improve data quality and reduce costs. Some research progress has already been achieved in eSource technology [4-6] in relatively large projects [7-9]. The review by Garza et al [5] included 14 studies detailing recent advances in eSource technology in clinical research. In total, 57% (8/14) of studies described single-site, single-EHR system implementation; 67% (4/6) of multisite studies were part of the same pilot study (EHR4CR European Pilot), a collaborative initiative across multiple European countries. Owing to the sensitivity of medical data, the variety of suppliers of medical information systems, and the low interoperability between medical systems, there is no similar eSource-related project in China. Therefore, it is common to use manual transcription data to conduct clinical research in China.

Real-world data (RWD) are data related to the patient health status and the delivery of health care that are routinely collected from a variety of sources [10]. A real-world study (RWS) collects RWD in a real-world environment and obtains real-world evidence (RWE) for the use value and potential benefits or risks of medical products through analysis [11]. Global regulatory agencies have issued a series of RWE-related guidelines, and researchers in different fields have shown interest in using RWD to conduct clinical research. Despite the availability of many relevant guidelines, various challenges in the use of RWD persist, such as inefficient data collection, lack of data quality control, and diversification of data standards and data compliance [12]. In China, data in the medical system are limited to the local area network, and external access and data sharing cannot be performed. Owing to the inability to coordinate hospitals’ concerns about the privacy of patients’ medical data and the needs of researchers for data transparency, the transformation and upgrading of existing medical system suppliers cannot meet the requirements of clinical research. The use of eSource technology in RWS is expected to solve the challenges of data collection efficiency and data quality, thereby reducing the cost of relying on manual transcription data to conduct research.

### eSource Record Project

In 2019, the China National Medical Products Administration established the Hainan Boao Lecheng Medical Tourism Pilot Zone as a pilot base for RWS, allowing domestic citizens to use global innovative products in China without first obtaining domestic market approval. Collecting and properly analyzing the RWD generated from patient visit data in Boao after using innovative medical products can help generate RWE that can be used for further domestic market approval. In 2020, the Hainan Real World Research Institute launched an eSource record (ESR) project to form an integrated solution and tool for hospital RWD collection, governance, and management. In this project, we completed the design and development of ESR software. The original intention of ESR was to provide a general tool that can implement RWS, thereby improving the implementation efficiency and quality of RWS research. Considering the need to develop many functions for old and underdeveloped medical supply systems to achieve automatic data capture and traceability of research data and to address the role of regulatory agencies and contract research organizations (CROs), we designed the ESR tool to meet the functional needs of clinical research. ESR tools can act as a bridge to connect EMR and EDC systems to achieve eSource technology. ESR was developed by a vendor to be used as a cost-saving method for conducting an RWS for sponsors.

After completing the development of the ESR tool, we tested and evaluated it in an RWS project in the Hainan Boao Lecheng Medical Tourism Pilot Zone. In a preliminary prospective study [13], we selected a cosmetic medical device project to evaluate the effect of ESR software on data collection and transcription. After completing the first evaluation of the ESR tool in the actual RWS project, we decided to expand the evaluation environment from simple RWS projects to more types of projects.

### This Study

As previously used medical esthetics research projects are relatively simple [13], we selected a completed ophthalmic femtosecond laser device project for retrospective data extraction and evaluation. Our team recently published a paper in the PharmaSUG China 2022 conference that introduced and evaluated a standardized method to convert source data to electronic case report form (eCRF) data in a real-world ophthalmology study [14]. This conference paper focused on the performance of natural language processing (NLP) in different types of data extraction. The accuracy of the data standardization method used in this study was 98.6%, but eCRF data completeness remained at 23.9% [14]. Through the case study, we summarized 2 key problems during the process of data transformation from real-world source data to eCRF data, namely, the lack of research-relevant source data and the complexity associated with the standardization of unstructured source data [14].

The purpose of this study was to evaluate the data transcription efficiency and quality of ESR software in retrospective studies to verify the feasibility of using eSource software as an alternative to traditional manual transcription of data in RWS projects. The ESR tool is currently in the exploration and evaluation stage; it is not yet fully mature and has not been pushed to the market for application. The contribution of this study was to provide more cases that use ESR tools in RWS projects to improve the design of tools and provide practical experience in exploring the application of eSource technology in China.

## Methods

### ESR Application Scenarios in the Hospital

ESR software links the EDC and EMR systems. The ESR system needs to receive the document format used in the EMR system and the reporting table field of the case in the EDC system and then convert the research field in the EDC system into writing suggestions in the EMR system document and send it to the EMR system. This strategy conforms to clinical doctors’ routine writing habits and data needs in research and increases the interoperability between systems. More details on the ESR software are available in a previous study [13].

The deployment of the ESR system in the hospital ensures the safety of the medical data. The deployment plan is described below and mainly involves two aspects:

1. The source data of various paths are required for integrated research to form a copy of the certificate. The overall theoretical framework is written back to the EMR system, according to whether the source data supplemented by the ESR are divided into 2 schemes:
  Plan A: ESR supplementary source data do not write back to the EMR system. The data of the health information system of the hospital move in one direction to the ESR system, and no other interaction is generated. In this mode, only data transmission interfaces from the clinical data repository of the hospital (EMR, laboratory information systems, and picture archiving and communication systems) to the ESR need to be established. The research source data required by clinicians only selected the research source data for ESR (excluding the full data of the patient). ESR forms a copy of the hospital source to copy the database for backup.
  Plan B: ESR supplementary source data are written back to the EMR system. This scheme was applied after the clinician used the ESR system to collect research data according to the research plan. This part of the supplementary data was selected to synchronously write back to the EMR system. Therefore, based on the content of regular EMRs for medical care, some ESR supplementary data are posted to realize the transformation of medical records into a more detailed scientific research medical record process and meet the data collection requirements of clinical research. This model can be used to develop more interfaces to interact with the EMR system.
2. The copy of the database is used to form a clinical research database (ie, ESR and EDC system docking).

When the data required by the institute are not suitable for the conventional diagnosis and treatment process, ESR can be used as a supplementary source data recording tool to prepare patients’ medical records through voice recognition technology and optical character recognition technology and to report adverse events. This function makes it convenient for doctors to efficiently complete supplementary collection of clinical research source data. When ESR is collected as a supplementary source, EMRs can be completed in a manner similar to conventional EMR systems in ESR. ESR can also follow the habits of doctors, configure structured meters for efficient data collection, and generate and record supplementary data required by clinical research.

### ESR-Based RWS Implementation Process

The research mode for implementing RWS on the basis of ESR software can be summarized in the following five steps:

1. Determining the research plan: first, as the premise of clinical trials, researchers must provide RWS research solutions and eCRF to collect data.
2. Configuring the traceability path of the eCRF in the EMR or other source files: eCRF topics can be associated with the EMR form to configure the traceability paths of the different eCRF topics. For example, demographic data in the eCRF can be traced back to the admission record form in the EMR.
3. Clinicians collect medical records and source data according to the prompts of medical record writing: routine medical records do not record certain necessary research-specific data such as scale scores. Therefore, after completing the eCRF traceability configuration, clinicians can design medical record writing prompts and rules for the eCRF that conform to clinical habits and meet their data collection requirements to cover the elements required for research and standardize the EMR recording process among different clinicians.
4. NLP technology intelligently extracts structured research data from the certified copy database into an eCRF: by connecting different medical systems that connect hospitals, the ESR system summarizes the source data of the hospital to form a copy of the database. The data outside the hospital and the source data of the EMRs recorded in ESR were entered into the certification copy database simultaneously. Using the NLP model, the ESR tool can capture data from free-text medical records. For structured data, ESR directly extracts data.
5. Data traceability and correction: ESR uses NLP technology to automatically extract data from the eCRF of the certification copy database in real time. This also supports the traceability of the source data for viewing these data. The clinical research coordinator (CRC) does not need to manually fill in the eCRF but can trace the source verification of the eCRF in the ESR. Through the traceability interface developed in the EDC system, the clinical research associate performs conventional source data verification and questioning work and sends queries to the ESR to remind clinicians to correct medical records.

### Pilot Case Selection

The femtosecond laser project is a prospective, single-group, and observational RWS of a femtosecond laser eye therapy system (CATALYS Precision Laser System [CATALYST]) for actual clinical diagnosis and treatment. The project was approved for marketing in 2021 and can be used in a typical case of ESR system performance evaluation in retrospective studies. CATALYST clinical research data were initially recorded in hospital information systems including EMRs and then manually entered into the eCRF rather than filling in the eCRF directly from eSource data. We selected 2 medical record forms (admission and surgical records) and the corresponding eCRFs for the research. The collected research data included the time spent on ESR system data transcription, correct rate of eCRF filling of the ESR system, and overall performance scores of different systems. The correct rate of eCRF filling referred to judging whether the filling value of the eCRF question was consistent with the source data according to the source data of the EMRs. After source data errors were excluded, if they were consistent, the question was completed correctly. By accessing log data from the EDC system previously used by the research project, all time spent from the start of the study to the end of the study could be counted. The time required for traditional manual transcription included the time required for data entry and correction by the CRC. The ESR software used NLP to automatically extract data from the EMRs in text form and used them to fill in the eCRF. When checking the source, the CRC needed to check the correctness of the fields that were filled in by the NLP system and needed to manually correct the incorrectly entered fields. The eSource transcription time included the CRC checking the correctness of the fields filled in by the NLP system and the time spent manually correcting incorrectly entered fields.

Times transcribed by the eSource software were timed using a stopwatch and manually recorded in Microsoft Excel. The data sources of the eCRF data variables and extraction method using the ESR system are listed in Table 1.

**Table 1:** Data sources for electronic case report form data variables and extraction methods using the eSource record system.

Research variable			Data sources		Source data record type		Extraction method
**Preoperative visit**							
	Subject information	Admission note (demographics)		Structured		Field mapping	
	Date of visit	Admission note (medical information)		Structured		Field mapping	
	Preoperative exam	Admission note (auxiliary examination)		Free text		NLP^a^ technology	
	Ocular history and medications	Admission note (history)		Free text		NLP technology	
	Uncorrected distant visual acuity	Admission note (specialist examination)		Free text		NLP technology	
	Best corrected distance visual acuity	Admission note (specialist examination)		Free text		NLP technology	
	Manifest refraction	Admission note (specialist examination)		Free text		NLP technology	
	Slit-lamp exam	Admission note (specialist examination)		Free text		NLP technology	
	Intraocular pressure	Admission note (specialist examination)		Free text		NLP technology	
	Biometry	Admission note (auxiliary examination)		Free text		NLP technology	
	Intraocular lens power calculation	Admission note (auxiliary examination)		Free text		NLP technology	
	Corneal topography	Admission note (auxiliary examination)		Free text		NLP technology	
	Cataract status	Admission note (history of present illness)		Free text		NLP technology	
	Dilated fundus exam	Admission note (specialist examination)		Free text		NLP technology	
	Ocular symptoms	Admission note (history of present illness)		Free text		NLP technology	
**Intraoperative visit**							
	Surgery date	Operative report (operation time)		Structured		Field mapping	
	Corneal incision type	Operative report (procedure)		Free text		NLP technology	
	Capsulotomy size	Operative report (procedure)		Free text		NLP technology	
	Lens removal	Operative report (procedure)		Free text		NLP technology	
	Phacofragmentation	Operative report (procedure)		Free text		NLP technology	
	Viscoelastic agent	Operative report (procedure)		Free text		NLP technology	
	Intraocular lens	Operative report (procedure)		Free text		NLP technology	
	Surgical medication	Operative report (procedure)		Free text		NLP technology	
	Anesthesia	Operative report (procedure)		Free text		NLP technology	
	Type of closure	Operative report (procedure)		Free text		NLP technology	
	Other surgical procedures	Operative report (procedure)		Free text		NLP technology	

^a^NLP: natural language processing.

### Ethics Approval

This study was conducted in accordance with the principles of the Declaration of Helsinki. Ethics approval was obtained from the Peking University Institutional Review Board (number IRB00001052–21081). Patient data were anonymized in accordance with the standards of clinical trials.

### Implementation Process

We configured the project in the development environment of the ESR system according to the research protocol and eCRF information from a previous project. Ophthalmic clinicians and CRO Company members who were residents of the department and had participated in numerous RWS projects were invited to participate in our testing assignment. Clinicians screened the information of all previous patient admissions in the hospital’s EMR system for the project. After exporting the basic hospitalization information of 90 patients, the EMRs of 30 patients were randomly selected according to the number of hospitalizations required to test the ESR system. The admission notes and operative report document content exported from the EMR system were copied into the ESR system to generate the same patient records. Using the medical record writing template, clinicians processed and generated 30 text corpora using fictitious information to train the NLP model. During the process of generating test medical records, clinicians could explore and experience different data input methods for the ESR system, such as voice input and optical character recognition. According to the annotation guidelines, 20 text corpora were annotated by the medical specialists. The technicians trained the NLP model using the annotated text, generated predictions for the remaining 10 patient records, and manually corrected the extracted fields to achieve model optimization.

### Data Standard Conversion Process

The EMR and EDC systems transmit data to the ESR system through the data standards of Health Level 7 Clinical Document Architecture and Clinical Data Interchange Standards Consortium (CDISC) Operational Data Model, respectively. The core of the data conversion process is to formulate text data labels on the basis of the most simplified data model, improve the efficiency of the NLP algorithm, and optimize the interoperability of clinical data models and the standard term library required by auxiliary extraction research. This process includes the five steps listed as follows [15]. The CDISC model used in ophthalmology is presented in Multimedia Appendix 1:

1. Send an eCRF to the ESR system from the EDC and send medical records from the EMR to the ESR system: the source data collection module of the ESR system is responsible for the EMRs and the collection of source data. The data transcription module of the ESR system is responsible for positioning the eCRF field to capture the text segment of the source data and to fill in the eCRF.
2. Research data collection models and the generation of labels: structural data are first mapped to the Observational Medical Outcomes Partnership model and then mapped to the CDISC model. Nonstructured data do not have a wide range of intermediate layers; they are directly converted to the CDISC model without considering the Observational Medical Outcomes Partnership model. The process of converting nonstructured data into research data is used to comment on and extract related content using the NLP model.
3. Extraction of model training and entity and entity relationships: in terms of physical extraction, a Chinese entity identification model, Bidirectional Encoder Representations from Transformers + Bidirectional Long Short-Term Memory Networks + Conditional Random Field, was adopted.
4. Generating a special research term: the dedicated term database is a mapping library between the actually extracted terms and standard terms in the indicator.
5. Standardization before entity extraction before filling in the eCRF: the output of the NLP model mainly includes the relationship table between all extracted label values and entities.

### Data Conversion Metadata

**Figure 1:**
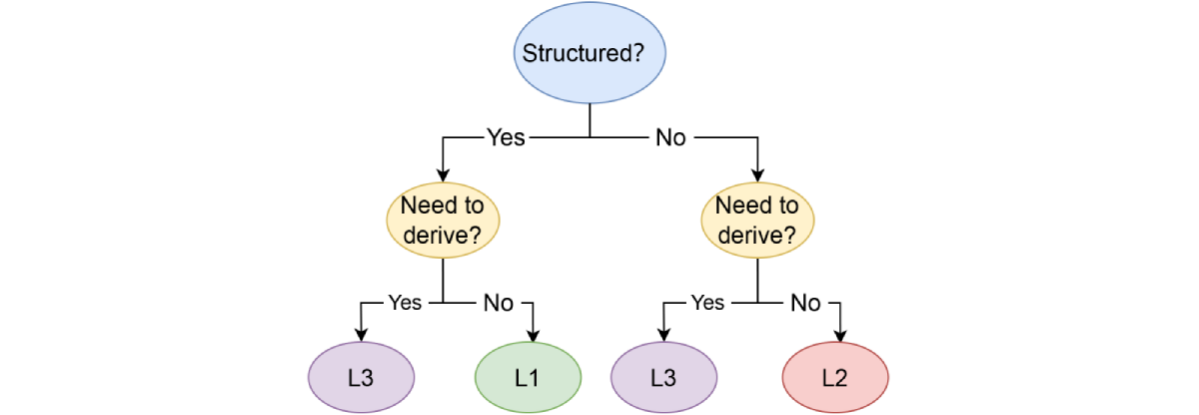
Data conversion classification method.

The data conversion from source data to CRF fields includes the conversion of both structured and unstructured text data. The details of this step are provided in previous papers by our team [14]. The classification of the data conversion methods can be seen in decision trees (Figure 1). Several data conversion examples are provided in Multimedia Appendix 2.

The first condition is whether the source data are structured or based on text. For surgery time fields (L1 category conversion), the structured source data can be directly used and converted into a standard time format. If the first condition is not met, it is necessary to use data transformation of the L2 category. NLP is used to extract the entity from the text. When extracting the entity, it is necessary to build an extraction rule such as using regular terms to extract related texts. To fill in a single research field, multiple entities must be extracted to determine which entity corresponds to the field in the report form. Therefore, after entity extraction, the output rules must be defined to extract field-related content.

After the physical extraction, regular extraction and term matching were performed. However, doctors’ use of diagnosis and treatment has not been standardized. They often need to add unrecognized terms to existing standard terms. The dictionary adds costs such as labor costs and employees who need clinical knowledge to identify the term in the text and the relation of the term to the corresponding standard terms. When constructing the output rules, information technology and clinical knowledge experts must capture the habits of the physician and write rules that can be summarized. In retrospective studies, establishing rules is the most cost-effective job, because doctors usually change their descriptions.

The second condition in the decision tree is whether the field must be derived to obtain the relevant content of the field, which is classified as the data conversion method of L3. Derivative data can originate from text or structural data. Structural data can be used in simple derivative algorithms. For example, the age field can use the extracted birthday field to derive the age of a subject. Data derived from text types must be treated as structured and derived fields. Taking the history of the disease as an example, it is necessary to derive fields after the extraction entity, and the output rules are judged. Here, the derivative algorithm is merged with the output rules as follows: (1) if (Past History-Regular) OR (Previous History Disease-Judgment) has nothing to do with (Negative Words), then output “Yes”; (2) if (Past History-Regular) OR (Previous History-Judgment) is related to (Negative Words), then output “No.” L3 fields sometimes need to use multiple outputs to derive research field content. For example, the field can include whether there is a discovery other than the specified scope of research. It is necessary to first exclude the output within the specified range to address other findings. L3 data transformation should not involve inferred or subjective judgment such as determining whether the event is a significant adverse event.

### Rating Scale

Usability includes effectiveness, efficiency, and overall user satisfaction. The System Usability Scale (SUS) is used to evaluate system usability [16]. The cross-industry average system availability scale score is 68, so this value is considered the threshold of acceptable availability. More details on SUS scores are available in previous studies [13]. The questionnaire consisted of the following 2 parts: participant information and SUS evaluation of the EMR, EDC, and ESR systems. In the questionnaire, the ESR system’s SUS score was the basic required question. For clinicians, the SUS score of the EMR system had to be completed simultaneously; for CRO company members, the SUS score of the EDC system had to be completed simultaneously.

### Data Analysis

Descriptive statistical methods were used to analyze the population participating in the questionnaire and their SUS scores. The data analysis software used in this study was Python (version 3.7.11). The Python *tableone* package (version 0.7.10) was used to generate demographic information for the questionnaires [17]. Cronbach α was used to evaluate the reliability of SUS. The Python *Pingouin* package (version 0.5.2) was used to calculate Cronbach α. A general acceptable range of Cronbach α is a value ≥.70 [18].

## Results

### eCRF Data Transcription Time

Admission notes and operative reports corresponded to the preoperative and intraoperative visit points of the eCRF, respectively. In the traditional method, the total entry time for the 51,424 fields at these 2 visit points was 11,738.85 minutes, that is, the average entry time for each field was 13.7 seconds. The eSource method required 6100 seconds for 2400 fields, which corresponded to an average entry time of 2.5 seconds per field. Therefore, the eSource method can save 11.2 seconds, that is, an overall time savings of 81.8%. The results are presented in Table 2.

**Table 2:** Data transcription times for the electronic source method (unit: seconds).

	Total number of eCRF^a^ fields, n	Total time (s)	Time spent per patient (s), mean (SD)
Admission notes	1890	5173	172.4 (15.0)
Operative reports	510	927	30.9 (4.8)
Total	2400	6100	203.3 (16.9)

^a^eCRF: electronic case report form.

### eCRF Data Transcription Quality

When using the traditional method, among the 51,424 fields entered, 47,991 fields were entered correctly, and the correct rate of entry was 93.32%. In the eSource method, the total correct entry rate was 98.17% (2356/2400). Using the eSource method for data extracted by NLP to fill in the wrong fields mainly focused on “slit-lamp examination lens” and “eye symptoms.” The main reason was that some described words were not in the dictionary of the NLP model, so they were not recognized. The results are presented in Table 3.

**Table 3:** Data transcription quality of the eSource method.

	Total number of eCRF^a^ fields	Fields filled in correctly by NLP^b^, n (%)	CRC^c^-corrected fields, n (%)
Admission notes	1890	1849 (97.83)	41 (2.17)
Operative reports	510	507 (99.41)	3 (0.59)
Total	2400	2356 (98.17)	44 (1.83)

^a^eCRF: electronic case report form.

^b^NLP: natural language processing.

^c^CRC: clinical research coordinator.

### System Performance Evaluation Questionnaire

Questionnaires were sent to 15 individuals participating in the femtosecond laser project test, and 15 questionnaires were received for a response rate of 100%. The characteristics of the participants of the questionnaire survey are presented in Table 4. In terms of usability, the average overall SUS scores of the EMR, EDC, and ESR systems were 50.3 (SD 21.9), 51.5 (SD 14.2), and 63.0 (SD 11.3; CRO experts: 69.5, SD 11.5; clinicians: 59.8, SD 10.2), respectively. The Cronbach α for the SUS items of the EMR, EDC, and ESR systems were 0.591 (95% CI −0.012 to 0.903), 0.588 (95% CI −0.288 to 0.951), and 0.785 (95% CI 0.576-0.916), respectively.

**Table 4:** The characteristics of the population participating in the questionnaire.

Items			Total (N=15)		CRO^a^ experts (n=5)		Clinicians (n=10)
**Sex, n (%)**							
	Female	9 (60)		5 (100)		4 (40)	
	Male	6 (40)		0 (0)		6 (60)	
Age (years), mean (SD)			27.3 (4.4)		27.8 (1.5)		27.1 (5.4)
**Profession, n (%)**							
	Clinical research associate	1 (7)		1 (20)		0 (0)	
	Clinical research coordinator	4 (27)		4 (80)		0 (0)	
	Clinician	10 (67)		0 (0)		10 (100)	
**Highest level of education, n (%)**							
	College degree and below	3 (20)		0 (0)		3 (30)	
	Undergraduate	4 (27)		3 (60)		1 (10)	
	Postgraduate	8 (53)		2 (40)		6 (60)	
**Experience in the medical field, n (%)**							
	1-3 years	7 (47)		2 (40)		5 (50)	
	≥10 years	1 (7)		0 (0)		1 (10)	
	<1 year	3 (20)		0 (0)		3 (30)	
	4-6 years	4 (27)		3 (60)		1 (10)	
**Frequency of EMR^b^ system use, n (%)**							
	Regular	2 (13)		0 (0)		2 (20)	
	Not applicable	7 (47)		5 (100)		2 (20)	
	Occasional use	1 (7)		0 (0)		1 (10)	
	Use every day	2 (13)		0 (0)		2 (20)	
	Frequent use	3 (20)		0 (0)		3 (30)	
**Frequency of EDC^c^ system use, n (%)**							
	Regular	1 (7)		1 (20)		0 (0)	
	Not applicable	10 (67)		0 (0)		10 (100)	
	Use every day	3 (20)		3 (60)		0 (0)	
	Frequent use	1 (7)		1 (20)		0 (0)	

^a^CRO: contract research organization.

^b^EMR: electronic medical record.

^c^EDC: electronic data capture.

## Discussion

### Principal Findings

This study provides specific examples of the use of ESR software in ophthalmology equipment to transform RWD into research data. Similar to previous case results from prospective studies [13], we found that the eSource approach was superior to manual data transcription in terms of data transcription quality, indicating that the improvement in data transcription efficiency provided by ESR systems does not sacrifice data quality. In the practice of the project, we found that although data transformation had good accuracy, more effective ways to improve the completeness of research data and implementation efficiency were lacking. Retrospective research typically does not use data standards before and during collection, resulting in difficulties in the process of data standard conversion in the later period. Therefore, data cannot be appropriately applied to eCRF, and information may be lost. This limitation explains why the data collection method used in prospective research based on research data standards is very important because it may directly affect the quality of the data from the starting point of data collection, thereby achieving standard conversion from RWD to clinical research data.

The main problems of low completeness include the lack of content specified in the description provided by the doctor recording the disease, lack of standard expression methods, and logic of describing clinical events. The process of development and transformation models must also be optimized, because they can only modularize a small number of fields but do not classify the same type of data, resulting in low development efficiency. In addition, the decrease in development efficiency is mainly because the clinical events described by doctors and the information required by the study are difficult to match. The overall data conversion process is long, and a real-time record of the details of all conversion processes and timely feedback on the quality of the data are unavailable. Therefore, the standardization of the doctor’s record, application of the data model to reduce the repeated transformation of the research field, details of the process of data transformation, and quality of the feedback data are necessary to optimize completeness and efficiency.

After summarizing the problems encountered, we proposed some possible solutions. First, the problem of the lack of matching of the research term with doctors’ habits may be solved by collecting commonly used terms to expand the coverage of the term and match the term using more automated approaches. Second, regarding the differences in context descriptions between doctors, some clinicians have proposed the use of recommended texts to promote the consistency of clinical event descriptions. Third, the lower development efficiency caused by the differences between fields may be improved by implementing suggestions for using data models. Finally, the problems of timely recording, data standardization, and feedback data quality may be solved by establishing source data management platforms to strengthen the source data and transparency of the data standardization process.

In retrospective case studies, the rate of complete data extraction is affected by the degree of consistency and vocally described by doctors. The solution at the time was to send the recommended text to the hospital EMR through ESR to strengthen the consistency of the physician’s description. However, from the perspective of experience, doctors still use different expressions for research data and efficiency cannot be improved. Therefore, technicians should use the NLP algorithm to extend the extraction rules and lists effectively. The improvement in the efficiency of artificial intelligence technology was attributed to the inclusion of each sample in the learning sheet of the model. In addition, the operating threshold during development was low and the efficiency was high. As long as the technical personnel responsible for the medical information can identify the entity and entity relationships in the text content, the model can automatically learn.

In a retrospective research environment, semantics allows researchers to affect the direction of data collection. After linking the hospital EMR documents and research data, clinical researchers and medical experts used research data models to generate text suggestions. When recommended terms are generated, researchers must consider methods that facilitate doctors’ use of the standard of research employed by the research terms to enable the collection of research data. According to the needs of field-specific terms for research, auxiliary instructions must be added to the prompt of medical records. However, if a relatively large standard library is needed, such as the Eleventh Revision of the International Classification of Diseases, we expect that the term query mode will be switched during the writing process such that the original terms and selected standard terms can be recorded. Therefore, this method can collect conventional terminology and match standard terms.

When applying ESR to real-world ophthalmology studies, we believe that the following conditions must be met: (1) first, specific research protocols and eCRF exist, and the research design is prospective research; (2) researchers must design a recommended text template for hospital EMRs according to the requirements of the research data, thereby promoting the standardization of data records; and (3) modules covering surgical medication, eye symptoms, additional surgery, and surgery have many prespecified events, such as whether star-shaped eye symptoms are observed. These events are rarely recorded in the research data. If technicians negotiate with the researchers in advance, the content that is not included may represent the incident or the researchers must clearly describe the content, which will improve the completeness of the eCRF data.

Similar to the EHR (hospital or clinic) SUS scores [19,20], we found that the hospital EMR SUS scores were also <60 points, which is the F level. In terms of system usability, the SUS score of ESR software obtained in this study was 63. Our research found that in hospital EMR and EDC, the SUS Cronbach α value was <.6. According to the classification of Cronbach α [18], a result ≤.6 indicates poor reliability of the hospital EMR and EDC SUS. The cause of this phenomenon may be related to the lower number of participants in our survey. However, the Cronbach α of the ESR SUS was >.7, which is acceptable. This further increases our confidence in ESR SUS scores.

The Duke University Clinical Research Institute introduced the RADaptor tool [21,22] as a solution to improve the efficiency of clinical research. The RADaptor tool acts as an intermediate plug-in to connect the EHR system (Epic) and the eCRF of REDCap (Research Electronic Data Capture; Vanderbilt University) software. The study by Nordo et al [22] used the RADaptor tool for case validation and evaluation and showed that this tool outperformed the traditional manual data transcription process. Unlike ESR systems that can be used for RWS, the current scope of the RADaptor tool is limited to single-site registration data. Following the eSource initiative of TransCelerate in 2016 [23], the latest work has now transitioned to the Health Level 7 Project Vulcan Fast Healthcare Interoperability Resources Accelerator [24]. The Vulcan project aims to help health care researchers more efficiently acquire, exchange and use, and translate data in clinical research using its widely recognized data exchange standard.

### Limitations

In terms of limitations, our study was limited by investigator selection and representativeness, similar to previous studies evaluating RWS projects on medical esthetics [13]. Furthermore, we were unable to obtain authorization to deploy the ESR system in the hospital intranet at the outset because of the need to demonstrate the value of the ESR software in an ophthalmology-based RWS project to hospital administrators. Therefore, we did not select all cases for evaluation but only sampled some cases to reduce the time spent transferring medical records from the hospital EMR system to the ESR system. Finally, the premise of this study is that the necessary fields for the study have been recorded in the previous CATALYST study medical records. Therefore, the extraction effect of ESR software largely depends on the method of scientific medical records, which is why we advocate project-based RWS. A homogenized medical record template will facilitate the standardization of terminology records in prospective studies, thereby reducing the burden of late NLP technology extraction.

### Conclusions

In ophthalmic RWS, the eSource approach based on the ESR system can replace the traditional clinical research model that relies on manual transcription of data. On the basis of this specific case, we provide experience in applying eSource technology to the transformation of RWD into research data. Follow-up research will focus on the deep functional integration of ESR and EMR systems to cope with complex research projects and optimize the RWS implementation process on the basis of the eSource approach.

## Appendix

Multimedia Appendix 1 Clinical Data Interchange Standards Consortium Model used in the ophthalmology field.

Multimedia Appendix 2 Several data conversion examples.

## Abbreviations

CATALYST: CATALYS Precision Laser System
CDISC: Clinical Data Interchange Standards Consortium
CRC: clinical research coordinator
CRO: contract research organization
eCRF: electronic case report form
EDC: electronic data capture
EHR: electronic health record
EMR: electronic medical record
eSource: electronic source
ESR: electronic source record
NLP: natural language processing
REDCap: Research Electronic Data Capture
RWD: real-world data
RWE: real-world evidence
RWS: real-world study
SUS: System Usability Scale

## Data Availability

The data sets generated and analyzed during this study are available from the corresponding author upon reasonable request.
