# Supplementary material for: Electronic Source Data Transcription for Electronic Case Report Forms in China: Validation of the Electronic Source Record Tool in a Real-world Ophthalmology Study
Source: JMIR Form Res. 2022 Dec 16;6(12):e43229. doi: 10.2196/43229 (PMC9804087; doi:10.2196/43229)
Supplement: Multimedia Appendix 1 [file formative_v6i12e43229_app1.pdf]

The CDISC model used in the ophthalmology field.

**DM （Demography）**						
DMSUBJID	BRTHDAY	AGE	SEX	ETHNIC		
123456 （Subject）	x	x	x	x		
**CM（Combination therapy）**						
CMTRT	CMINDC （Indication of medication）					
History of medication	x					
**DS（Subject status）**						
DSTERM	DSYN （Yes NO）	DSSTDAT （Date）				
Informed consent	x	x				
**OE （Ophthalmology）**						
OETEST	OELAT （Location of the eyes）	OERES （Result）	OEPERF （Perform）	OEDAT （Date）	OERIND（Whether significant）	OEMETHOD （Method）
UCDVA	x	x				

BCDVA	x	x				
manifest refraction			x	x		
D Sphere -OD	x	x				
D Sphere -OS	x	x				
D Cylinder -OD	x	x				
slit-lamp exam			x	x		
Lids/Adnexa -OD	x	x			x	
Lids/Adnexa -OS	x	x			x	
intraocular pressure	x	x				x
Axis length -OD	x	x				
Axis length -OS	x	x				
Lens Power -OD	x	x				
Lens Power -OS	x	x				
A-Constant -OD	x	x				
corneal topography			x			
cataract status -OD	x	x				

cataract status -OS	x	x				
dilated fundus exam-OD	x	x			x	
dilated fundus exam-OS	x	x			x	
Vision decreases -OD	x	x				
Vision decreases -OS	x	x				
Optical/Visual -OD	x	x				
Optical/Visual -OS	x	x				
Entopic phenomenon -OD	x	x				
Entopic phenomenon -OS	x	x				
Sensation/Other -OD	x	x				
Sensation/Other -OS	x	x				
**PR（Operation）**						
PRTRT	PRTRTCMP（Whether to complete）	PRDAT（Date）	PRCAT（Classify）	PRDSTXT（Describe）	PRLAT（Location of the eyes）	PRMETHOD（Method）
Corneal Incision	x	x	x	x	x	
Capsulotomy	x				x	x

Lens removal	x		x			
type of closure			x			
Preoperative examination of cataract surgery	x	x				
**DO（Device attribute）**						
DOTEST	DOORRES （Result）					
Artificial Crystal-Model	x					
Artificial Crystal- Lens Power	x					
**AG（Drugs used during operation）**						
AGTRT	AGOCCUR （Whether to execute）					
Antibiotic	x					
BSS Used	x					
IOP Reducing	x					
Miotic	x					

Mydriatic/Cycloplegic	x					
NSAID	x					
Steroid	x					
Anesthetic	x					
