# Supplementary material for: Electronic Source Data Transcription for Electronic Case Report Forms in China: Validation of the Electronic Source Record Tool in a Real-world Ophthalmology Study
Source: JMIR Form Res. 2022 Dec 16;6(12):e43229. doi: 10.2196/43229 (PMC9804087; doi:10.2196/43229)
Supplement: Multimedia Appendix 2 [file formative_v6i12e43229_app2.pdf]

Conversion of CRF Fields

CRF Field	Source Data	Conversion Route	L1 (Mapping)	L2 (Entity)	L2 (Entity Relation)	L2 (Output)	L3 (Derived)
Operation Time	2021/3/13 10:44	S>L1>T	18 digit standard date time				
UCDVA Left Eye Result	Eye Exam: UCDVA：OD：0.6，OS：0.08；BCDVA: OD: 0.6 OS: 0.4;	S>L2>T		1. “UCDVA”: (UCDVA) 2. “Left Eye”: (OS, Left Eye) 3. “Result”:(/^[-+]?[0-9]+[.]?[0-9]*([eE][-+]?[0-9]+)?$/)	1. “UCDVA” in front of “Left Eye”, both entities in a sentence 2. “Left Eye” in front of “Result”, both entities in a phrase	Output “Result”	
Has UCDVA been Performed		S>L2>L3>T					If “Result” is not null, then “Yes”, else “No.
